# Non-fucose level as a function of glycoenzyme transcription in a glycoengineered CHO cell line

**DOI:** 10.1186/1753-6561-9-S9-O5

**Published:** 2015-12-14

**Authors:** Sven Markert, Harald Wizemann, Christine Jung

**Affiliations:** 1 Pharma Biotech Development Fermentation, Roche Diagnostics GmbH, Penzberg, Germany

## Background and novelty

Antibody-dependent cellular cytotoxicity (ADCC) is one important mode of action for therapeutic mAbs in the field of oncology. It is strongly dependent on the glycan pattern of the Fc N-glycan: low core-fucose levels (= high non-fucose levels) typically result in an increased ADCC. There are several options to reach high non-fucose levels like the selection of the right host that expresses the desired pattern, in vitro glycosylation, use of glycosylation inhibitors, alteration of process parameters and generating glycosylation mutants with modified glycan synthesis activities by cell line engineering.

Within Roche Pharma we are working with CHO cell lines designed to produce therapeutic antibodies based on the GlycArt system. These cell lines contain in addition to the recombinant gene for the therapeutic monoclonal antibody (mAb), also recombinant genes for two glycosyltransferases, N-acetylglucosaminyltransferase-III (GntIII) and mannosidase-II (ManII). As a result the CHO cells produce antibodies with a modified glycosylation structure characterized by a low-fucose Fc part. The selection system for the two glycosylation enzymes is based on the use of puromycin whereas for the mAB MSX is used.

## Experimental approach

During the scale up of a cell culture process for a late stage project we observed that the cell age might influence the non-fucose level and thus the ADCC of the recombinant monoclonal antibody negatively. To ensure a high product quality even at a high cell age we investigated the correlation between cell age and non-fucose level in more detail by identifying underlying mechanisms with focus on the glycosylation enzymes GntIII and ManII that are overexpressed in this cell line. For this purpose a method was established in collaboration with Roche Diagnostics to quantify the gene expression level of the glycosylation enzymes using RT-qPCR based on the RealTime ready technology.

At the beginning of the project a cell age study was conducted using shake flasks in serial culture mode to generate cells with different and especially high cell age. The cells were cultured under different selective conditions: (1) a combination of puromyin and MSX (2) only with puromycin, (3) only with MSX and (4) without selective pressure. Cells were frozen at different time points up to a cell age of 97 days. Afterwards a fed-batch experiment with all cell banks of different cell age and selective conditions simultaneously was run. The fed-batch experiment was conducted with our in-house developed robotic cell culture system that enables a fully automated workflow based on shaken multiwell plates [1].

## Results and discussion

The data from the cell age study verified the finding that the cell age negatively influences the non-fucose levels. We could show that the combination of puromycin and MSX stabilizes the non-fucose level at a high cell age up to 110 days whereas the use of puromycin or MSX alone provides only a slight stabilization. The cultivation without selective pressure resulted in the lowest non-fucose levels. Running the automated fed-batch experiment we could verify the results from the cell age study and we could also show that the results from the automated system are predictive for a bioreactor. To understand the role of the glycosylation enzymes in this context we quantified the gene transcription level of the recombinant glycosylation enzymes ManII and GnTIII.

Since a suitable RT-qPCR method was not available we developed a customized method based on the RealTime ready technology in collaboration with colleagues from Roche Diagnostics.

**Figure 1:**
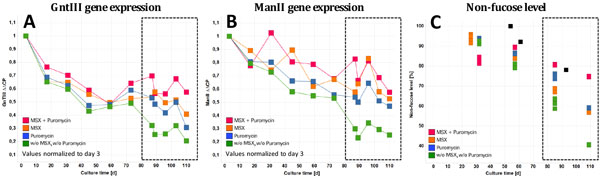
**(A) GntIII gene transcription data; (B) ManII gene transcription data; (C) Non-fucose level**. For (C): Colored squares represent data from automated cell culture system and black squares from 10.000 L bioreactor.

The mRNA levels of GntIII and ManII were measured over the course of the seed train study (shake flask) using the developed RT-qPCR method and related to corresponding glycosylation data of the mAb at the end of the fed-batch production run in our cell culture robotics facility (Figure 1). At high cell age a direct correlation between non-fucose level and GntIII gene transcription level could be shown, whereby the highest mRNA levels were obtained for the cultures that used the combination of puromycin and MSX. The absence of selective pressure resulted in the lowest GntIII mRNA levels and thus the lowest non-fucose levels. The correlation between ManII mRNA and non-fucose level is not as clear as seen for the GntIII however the stabilizing effect of selective pressure could be shown.

The stabilizing effect of selective pressure on non-fucose level as well as the direct correlation between GntIII mRNA and non-fucose levels could be confirmed with a second recombinant cell line.

Based on the results of this study cultivation recommendations regarding the in vitro cell age and the selection pressure for the seed- and inoculum-train of a production process could be provided. Also a direct correlation between selective pressure addition, GntIII and ManII transcription level and the non-fucose level for cells with a high cell age could be identified. The results of this study provide insight into the mechanisms of glycosylation of recombinant GntIII and ManII cell lines. Data from the robotics experiment closely mirrored the production scale.

Furthermore, a high throughput RT-qPCR method was developed based on the RealTime ready technology to monitor gene transcription based on CHO cells that was not previously available. This platform will be further expanded to be applied to more CHO genes.

## Acknowledgements

We would like to thank all colleagues who contributed to this work, namely M. Baumeister, S. Eppler, K. Graf, M. Hechler, A. Höfer, H.G. Ihlenfeldt, O. Kaiser, N. Matthei, K.C. Nabo H. Walch and M. Wiesinger from Roche Diagnostics, K. Diepold, A. Esterl, J. Gassner, M. Gräf, M. Haberger, J. Hoffmann, M. Leonhardt, C. Musmann, G. Pechmann, M. Radtke, F. Schache, C. Schuster, A. Strauch, M. Weichert and F. Weissbecker from Roche Pharma Biotech Development Fermentation and our management, J. Gabelsberger and W. Kuhne for their continuous support and helpful contributions.
